# Genetic Influence on Slope Variability in a Childhood Reflexive Attention Task

**DOI:** 10.1371/journal.pone.0130668

**Published:** 2015-06-23

**Authors:** Rebecca A. Lundwall, Jeffrey K. Watkins

**Affiliations:** 1 Psychology Department, Brigham Young University, Provo, UT, United States of America; 2 Neuroscience Center, Brigham Young University, Provo, UT, United States of America; Instituto de Higiene e Medicina Tropical, PORTUGAL

## Abstract

Individuals are not perfectly consistent, and interindividual variability is a common feature in all varieties of human behavior. Some individuals respond more variably than others, however, and this difference may be important to understanding how the brain works. In this paper, we explore genetic contributions to response time (RT) slope variability on a reflexive attention task. We are interested in such variability because we believe it is an important part of the overall picture of attention that, if understood, has the potential to improve intervention for those with attentional deficits. Genetic association studies are valuable in discovering biological pathways of variability and several studies have found such associations with a sustained attention task. Here, we expand our knowledge to include a reflexive attention task. We ask whether specific candidate genes are associated with interindividual variability on a childhood reflexive attention task in 9–16 year olds. The genetic makers considered are on 11 genes: *APOE*, *BDNF*, *CHRNA4*, *COMT*, *DRD4*, *HTR4*, *IGF2*, *MAOA*, *SLC5A7*, *SLC6A3*, and *SNAP25*. We find significant associations with variability with markers on nine and we discuss the results in terms of neurotransmitters associated with each gene and the characteristics of the associated measures from the reflexive attention task.

## Introduction

Interindividual variability occurs in a variety of human behaviors, including how we pay attention. Such variability may arise because it takes effort to maintain focus while attending to certain tasks, and there are periodic lapses in the ability to sustain that effort [1]. Variability measures are commonly included on sustained attention tasks such as the Continuous Performance Test (CPT) [2] and the Sustained Attention to Response Task (SART) [3]. There are also elements of effort over the course of a reflexive attention task.

The variability captured during a reflexive attention task is an important component of attention (representing an individual’s ability to ignore distracting stimuli that “pull” attention in reorienting of the brain’s resources). It can be measured using a modification of Posner’s orienting task [4]. In these tasks, stimuli often newly appear, have a relatively salient color, or involve motion [5–7]. Like many human behaviors, the ability to ignore distractors varies from time to time. Some participants will display more variability than others.

**Fig 1:**
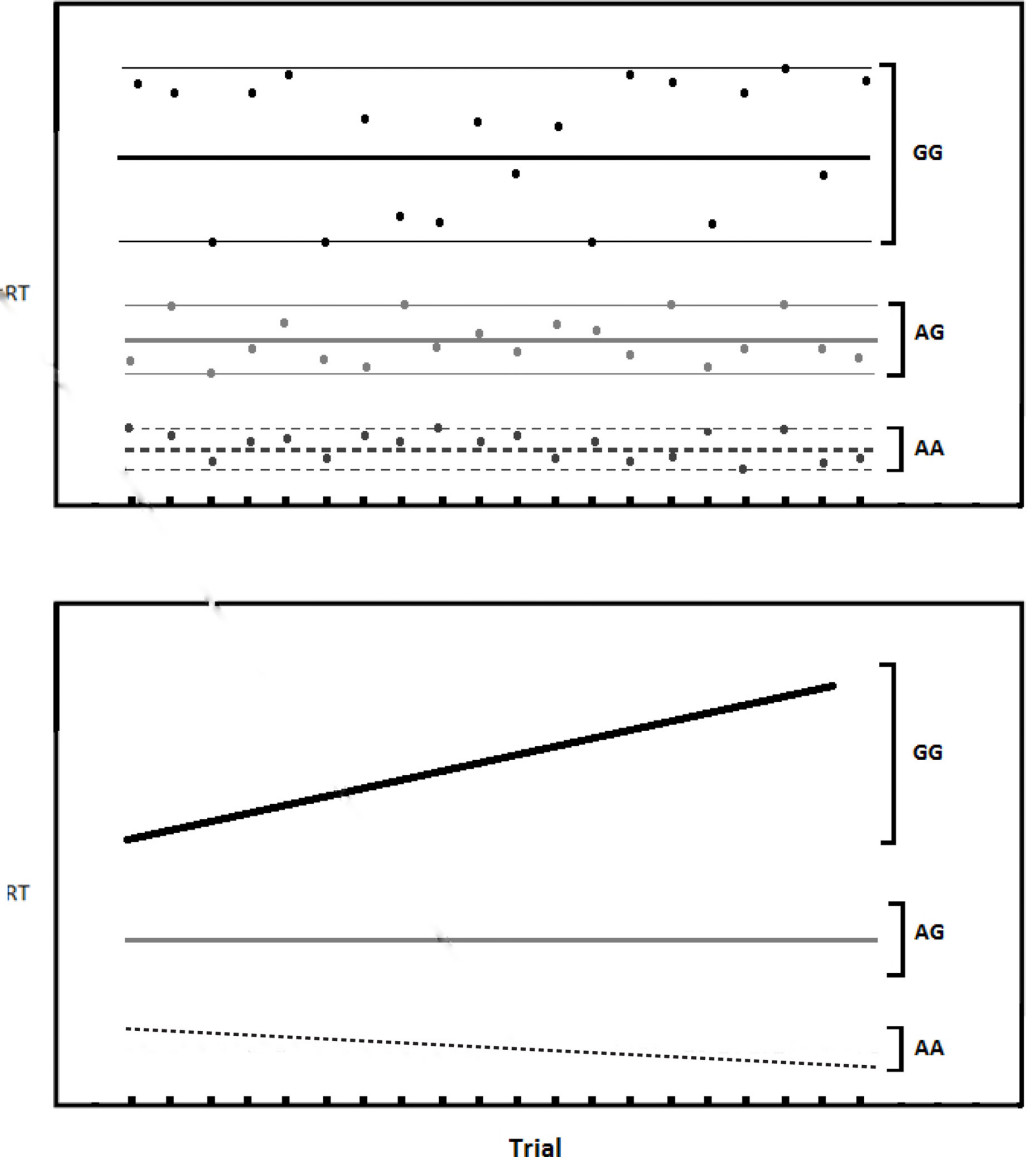
Hypothetical illustration of the two types of variability addressed in this paper. *Top*. Moment-to-moment variability showing more variability for the GG genotype and less for the AA genotype. *Bottom*. Slope variability showing increase RT over the course of the task (GG genotype) and decreasing RT (AA genotype). It is possible for subjects to have both types of variability. In this paper we examine slope variability.

While moment-to-moment variability (see the top portion of Fig 1) is commonly measured, we are interested in the less commonly measured *slope variability*, which refers to the change in a participant’s RT over the course of the task. It might be observed, for example, that early task RTs are faster than later task RTs (see the bottom portion of Fig 1). This might occur because once-salient stimuli become habituated (less-novel). Alternatively, some participants may start a task with slow RTs and speed up as the task progresses, which might happen if learning is improving performance. RT variability seems to be as important in examining individual differences as mean RT [8–10]. The tendency to speed up or slow down might index certain aspects of the individual’s overall ability to pay attention.

While slope variability is not well studied in reflexive attention tasks, genetic associations are particularly lacking. Genes make proteins, including neurotransmitters, receptors, and enzymes that catabolize neurotransmitters. Therefore, it is plausible that the biological pathway between the brain and behavior (such as attending) will include neurotransmitters whose availability make neural transmission more or less efficient. Two types of genetic markers are single nucleotide polymorphisms (SNPs) and variable number tandem repeats (VNTRs). A SNP is a variation at a single point (i.e., a single nucleotide) in the genetic sequence. A VNTR is a variation in the number of copies of a short string of nucleotides. The nucleotide (for SNPs) and number of copies of a repeat (for VNTRs) is inherited from parents.

Although few studies have examined the influence of genetic markers on reflexive attention task variability at all (either moment-to-moment or slope), several studies have found plausible evidence for the link between specific genetic markers and variability on sustained attention tasks. For example, one hypothesis is that genetic contribution to RT variability occurs via a dopamine deficit. This hypothesis is supported, in part, because those with ADHD have been found to have reduced dopamine levels [11] and medication that increases dopamine availability is helpful in treating ADHD [12]. But there are also subtle effects on individuals in the general population. For example, Stefanis, van Os [13] found that a Val allele on *COMT* (which catabolizes dopamine) is associated with increased RT variability on the CPT in the general population. This is significant because it indicates that research that may lead to pharmacological treatments can begin by studying very specific deficits (endophenotypes) [8, 14] in the general population.

In addition, some genes have been associated with slope variability on sustained attention tasks. For example, Thakur, Sengupta [15] found significant associations between *SLC6A2* (which codes for the norepinephrine transporter) and Hit RT Block change of the CPT (which measures slowing over the course of the task). The risk allele was overtransmitted to individuals with higher scores (i.e., more RT slowing). *SLC6A2* is responsible for reuptake of norepinephrine into the presynaptic membrane. Because norepinephrine is synthesized from dopamine the relative levels of these two neurotransmitters are related and this is indirect evidence that genotype can influence the slope of RT over the course of a task.

Another genetic hypothesis is that acetylcholine contributes to attentional variability. This hypothesis is supported, in part, because nicotine patches (which act on acetylcholine receptors) improve general attention in both schizophrenic patients and nonpsychiatric controls [16]. Findings with acetylcholine also have implications that may be used to further research into pharmacological treatments and practical interventions targeted to very specific attentional behaviors.

Finally, we examine genes that are associated with serotonin, which acts as a signal in brain development [17] and has been associated with social attention in infants [18]. Serotonin has also been associated with attention-deficit disorder generally [19, 20] and with both behavioral and cognitive impulsivity more specifically [21]. Of particular interest to this study, serotonin has been associated with increased RT variability [22–24]. Therefore, this neurotransmitter likewise appears linked with some aspects of attention.

Since evidence exists that links specific genes with interindividual variability on sustained attention tasks and with slope variability as well as moment-to-moment variability, research should be conducted to see if this link holds in reflexive attention tasks. We examine genes related to three neurotransmitters (acetylcholine, dopamine, and serotonin) which have already been associated with attentional variability and brain development. The genetic markers selected for the study are on 11 genes: *APOE*, *BDNF*, *CHRNA4*, *COMT*, *DRD4*, *HTR4*, *IGF2*, *MAOA*, *SLC5A7*, *SLC6A3*, and *SNAP25*. We studied these genes using slope variability on a reflexive attention task where we expected individual differences in the ability to maintain consistent RTs. Performance data on RT slope over the reflexive attention task was analyzed in relation to genetic data.

## Materials and Methods

### Participants

#### Recruitment

Participants were recruited after Institutional Review Board (IRB) approval from Rice University (IRB-Human subjects) and the University of Wisconsin-Madison (the Social and Behavioral Sciences IRB). Additional data analysis was performed under Institutional Review Board approval from Brigham Young University (the IRB for Human Subjects). All procedures were in accordance with the ethical standards of these universities and with the Helsinki Declaration of 1975, as revised in 2000. Written informed consent was obtained from the parents of all children and assent was obtained from children included in the study. Participants were recruited from 854 children (9–16 years old) for whom we had infant attention scores [25]. When they were 3–6 months old, the participants had been presented with a display with a moving bar in a field of static bars. An adult observer, blind to the side of the moving bar, made left-right judgments of the side of the infants’ looking. The data we have on the infants includes the percent correct (PC) compared to the actual side of the moving bar. We used infant PC the residual scores (representing variability to be explained after controlling for gestational age, sex, and birthweight) and these scores were used to determine which children to invite for computer testing in the lab. Data collected represent an oversampling of the upper and lower one-third of the distribution because we determined that these children most likely to represent children who might require intervention.

Of the 854 original participants, we excluded 77 children who could not be located 10–15 years after they were first tested and 21 who had a sibling in the study (to avoid genetic associations due to non-independence). Remaining children who lived in the Madison, Wisconsin, area and were in the upper and lower one-third of the infant distribution of residual PC scores were invited to visit the Waisman Center to complete the computerized attention task. Children who did not live in the Madison area or were in the middle one-third of the infant distribution were invited to participate by mail, however 14 children from the middle one-third also participated. Of the 756 invited to participate, 203 participated in-person and 129 participated by mail. This is a 44% participation rate, which is not unusual for a long-term longitudinal follow-up study [26–28], especially when there is no intervening contact over the 10 to 15 years. All children, whether they participated in-person or by mail, provided a saliva sample and their parents completed a questionnaire. The attention task completed by the 203 children available to travel to the Waisman Center is described below.

#### Exclusion criteria

Only the 203 participants who participated in-person were considered for the final analysis. However, children with an error rate over 40% (*n* = 5) were excluded. Two additional children were excluded for having a serious neurological disorder. Children whose parents (by their own definition) reported that their child had at least one non-white biological parent (*n* = 4) were excluded in order to help avoid population stratification (confounding due to a systematic difference in allele frequencies) [29]. Finally, two children were excluded for having uncorrected visual disorders. The data of these remaining 190 children are included in final analyses.

### Measures

**Fig 2:**
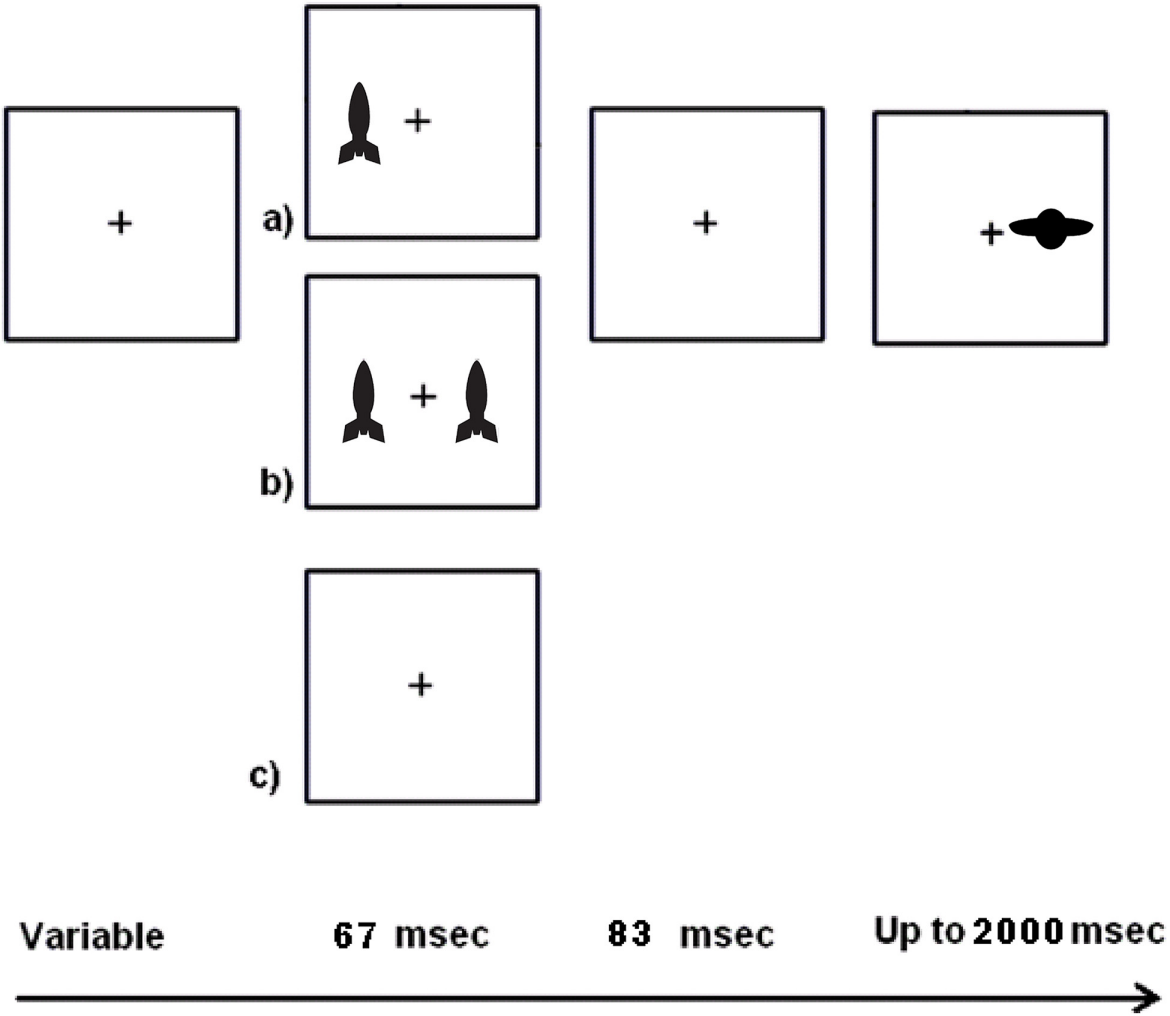
Schematic of the child spatial cueing (SC) task. The cue duration plus the gap duration (inter-stimulus interval) makes the stimulus onset asynchrony (SOA) 150 msec. The inter-trial interval varied from 600–3000 msec.

The spatial cueing (SC) task used in this study was a Posner-like reflexive attention task [4] and was similar to that used in our previous study conducted with adults [30]. The child version of the task was modified to engage the interest of children while still maintaining the ability to measure visual reflexive attention to suddenly appearing stimuli a (Fig 2). The stimuli we used, rockets and alien spaceships, were designed to be salient and attractive to children (in contrast with a sustained attention task, which has tedious stimuli by design).

Participants were tested in a darkened room on a 381 x 305 mm monitor with a 60 Hz refresh rate. Viewing distance was maintained at 57 cm with a chin rest. EPrime (Sharpsburg, PA) was used to present stimuli. Cues had an inner edge 7.0 deg from central fixation. Targets had an inner edge 5.7 deg from fixation. The task was designed as a game with friendly “earth” rockets and alien spaceships. Earth rockets flashed briefly (67 msec) and acted as cues. Children were told to ignore them as much as possible. After a brief gap (83 msec) an alien spaceship could appear. These were targets to which children were instructed to make a right or left key press (spatially mapped to the location of the target). The cues could be valid (i.e., appear near where the target would appear) or invalid (appear contralateral to the target). Fifty-percent of the cues were valid. Since this is chance, children were told that paying attention to the earth rockets would not help them. Participants are typically faster at responding to a target that is preceded by a valid pre-cue even though the stimuli presentation is too brief to depend on eye movement. There were also neutral cues, with one cue on each side of the display. This condition did not bias attention to either the left- or the right-side. The use of two different cue contrasts (faded and unfaded, described here as dim and bright, respectively) yielded seven measures (i.e., No Cue, Neutral Dim, Neutral Bright, Single Bright Valid, Single Bright Invalid, Single Dim Valid, and Single Dim Invalid). There were 24 trials of each condition intermixed and pseudo-randomly presented over the course of the task. There were also 12 catch trials with not target on which participants were instructed to withhold response. Only a participant’s correct trials were included in analyses (Table 1).

**Table 1:** Primary measures.

Primary Measure	Description
No Cue	No cue prior to target
Dual, both dim	Two dim cues (left and right) prior to target
Dual both bright	Two bright cues (left and right) prior to target
Single dim valid	Single dim cue ipsilateral to target
Single bright valid	Single bright cue ipsilateral to target
Single dim invalid	Single dim cue contralateral to target
Single bright invalid	Single bright cue contralateral to target

We also measured daytime sleepiness in children using the Epworth Sleepiness Scale-Children (ESS-C) [31]. The original version of the Epworth Sleepiness Scale (ESS) has eight items. All items are statements to which the participant is to respond “How likely are you to doze off or fall asleep in the following situations in contrast to just feeling tired?” The child version (ESS-C) has seven of the original items plus a replacement item. Instead of likelihood of falling asleep “in a car, while stopped for a few minutes in the traffic” the child version has likelihood of falling asleep while “doing homework or taking a test.” All questions have to do with falling asleep in daytime situations where people who are getting enough sleep are unlikely to fall asleep [31]. Parents completed the questionnaire for their children. We used sleepiness scores as a covariate in the statistical models.

### Genetic data

The markers under consideration were on 11 genes (i.e., *APOE*, *BDNF*, *CHRNA4*, *COMT*, *DRD4*, *HTR4*, *IGF2*, *MAOA*, *SLC5A7*, *SLC6A3 [DAT1]*, and *SNAP25*). These genes are related either to the availability of neurotransmitters such as acetylcholine, dopamine, and serotonin and to growth, brain development, attention, or general cognition. Multiple markers were used for each gene. These markers are SNPs and VNTRs. Risk alleles for some aspect of attention or cognition are known for 23 of the 39 markers.

Genetic data were collected from each participant using an Oragene-500 kit (DNA Genotek, Kanata, Ontario, Canada). Each kit collects approximately 2 ml of saliva, which can then be purified to obtain DNA. Nucleotides can be tagged with a fluorescent dye. Genotyping was performed using the GoldenGate assay on the BeadXpress system (Illumina, Inc.). Once the array had been visualized with the BeadXpress reader, wavelength and intensity values of the fluorescence were used to determine genotype. Allele detection and genotype calling were performed using GenomeStudio software v2011.1 (Illumina, Inc.). Genotype was coded as an ordinal variable with zero indicating no genetic risk, one indicating intermediate risk, and two indicating higher risk of attentional deficits.

### Statistical analysis

As described under Recruitment, we used a modified extreme group design including a limited number of participants from the middle of the infant PC distribution. This is an oversampling of the extreme ends of the distribution. Those in the extreme groups might need intervention in the sense that (if we could determine infant indicators of child difficulties) we could try to prevent infants from developing problems as children. However, we did not specifically try to recruit children who were having such problems because the traits we are interested in are likely normally distributed in the population. Using a specific endophenotype rather than a diagnosis to define groups is a genetically sound practice.

Statistically, by minimizing the within-group variance and maximizing the between group differences in the predictor variables it becomes easier to look at the relations between predictors and outcomes. Even though some researchers have discouraged the use of extreme group designs, the use of a continuous variable and an oversampling of the extremes is statistically justified [32]. We have data from 14 cases in the middle one-third of the infant distribution who are included in the analysis.

Prior to analyses, RTs were prepared as follows. Only RTs of correct trials were analyzed for genetic association. Correct responses were those for which the participant (1) selected the side on which the target appeared and (2) responded between 200 and 1000 msec after the target appeared. It is nearly impossible for a child to respond to a target in under 200 msec and so RTs lower than this were assumed to be anticipatory. Likewise, RTs longer than 1000 msec are quite slow for this type of task and were assumed to represent lapses in attention.

To examine slope we used a mixed (multilevel) model analysis. A multilevel model analysis is similar to a regression analysis but accounts for the nested nature of the data. We used this method because an intraclass correlation indicated that 31% of the variance came from the individual (level 2) data and we needed to take this into account when analyzing trial level (level 1) data. We entered all 39 markers, sleepiness, age, trial number, infant percent correct score, and the interaction between trial number and genetic marker as predictors of RT. However, because we were interested in slopes over the course of the task, we only examined output pertaining to the interaction between the genetic marker and trial number. Models for each of the seven outcome variables were tested separately using SPSS version 21.

## Results

### Descriptives

In this study, all SNPs were in Hardy-Weinberg equilibrium with all *p*-values greater than .36. This indicates that, given the minor allele frequency based on a Caucasian population, the observed genotypes are not significantly different from the expected genotypes.

Average error rate per child was highly variable (range 0% to 53.89%, *M* = 7.83%, *SD* = 6.64%), therefore, based on the distribution of error rates we excluded data from children with an error rate over 40% (*n* = 5). Of the children whose data were included, 50.92% were male. Their ages ranged from 9 to 16 years (*M* = 12.46, *SD* = 1.77).

Using only included participants, RTs ranged from 200 to 999 milliseconds (*M* = 390.90 msec, *SD* = 95.04 msec). Because RTs were somewhat skewed (skewness = 1.48, *SE* = 0.18), the logarithm of RT was used for statistical analyses so that the data met assumptions for normality. As expected, skewness was reduced, skewness = 0.04, *SE* = 0.18.

### Main analyses

We examined interactions between genotype and trial number using seven multilevel models predicting log transformed RT. As described above, multilevel modeling is similar to regression analysis but accounts for the nested nature of the data (i.e., trials can be attributed to individuals). We entered 39 genetic markers, sleepiness, age, infant percent correct score, trial number and the interaction between trial number and genetic marker as predictors of log transformed RT. The improvement of each model over a baseline model with no predictors was significant (for all models, χ^2^ [134, *N* = 29880] > 1934.70, p < .001), and all models passed Bonferroni correction. Because our primary question involved genetic associations with RT slope, we examined the interaction between genotype and trial number in predicting log transformed RT for each of seven outcome measures. Five of seven mixed multilevel models contained a total of 21 significant associations (involving 15 genetic markers; see Table 2). Following a brief overview, we will report each significant genetic association.

**Table 2:** RT Slope Variability Means

				0 Risk Alleles	1 Risk Allele	2 Risk Alleles
Genetic Marker	DV	Genotypes	*p*-value	Mean (SD) n	Mean (SD) n	Mean (SD) n
BDNF rs2203877	NB	CC;CT;TT*	0.04	-0.11 (.37) n = 46	-.10 (.41); n = 92	-.13 (.30); n = 52
BDNF rs6265	NB	AA;AG;GG	0.03	0.00 (.26); n = 10	-.12 (.34); n = 58	-.11 (.39); n = 122
DRD4 rs1800955	NB	TT; CT;CC	0.04	-0.11 (.32); n = 61	-.15 (.38); n = 86	-.03 (.41); n = 43
SLC5A7 rs3806536	NB	AA;AG;GG*	0.03	-0.04 (.32); n = 71	-.14 (.41); n = 95	-.16 (.33); n = 24
SLC5A7 rs4676169	NB	AA;AG;GG	0.03	0.02 (.40); n = 35	-.15 (.37); n = 102	-.10 (.33); n = 53
SLC6A3 rs2617605	NB	AA;AG;GG*	0.01	-0.07 (.41); n = 74	-.03 (.21); n = 2	-.13 (.34); n = 114
SLC6A3 rs2937639	NB	AA;AG;GG	0.02	0.02 (.37); n = 32	-.13 (.38); n = 96	-.14 (.35); n = 62
DRD4 rs1800955	No Cue	TT; CT;CC	0.03	-0.01 (.38); n = 61	-.17 (.45); n = 86	.08 (.80); n = 43
IGF2 rs734351	No Cue	CC;CT;TT*	0.03	-0.05 (.85); n = 33	-.01 (.39); n = 89	-.14 (.52); n = 68
SLC5A7 rs4676169	No Cue	AA;AG;GG*	0.01	.04 (.42); n = 35	-.15 (.47); n = 102	.03 (.70); n = 53
SLC6A3 3UTR VNTR	No Cue	5R/5R; 5R/6R;6R/6R	0.049	-0.24 (.60); n = 14	-.04 (.67); n = 60	-.06 (.45); n = 116
APOE rs7412	SBI	TT; CT;CC	0.03	0.62 (.02); n = 2	.02 (.45); n = 28	-.03 (.42); n = 160
SLC6A3 rs2042449	SBI	CC;CT;TT*	0.01	.00 (.46); n = 113	-.07 (.36); n = 68	.08 (.43); n = 9
SLC6A3 rs2617605	SBI	AA;AG;GG*	0.01	-.01 (.45); n = 74	.30 (.52); n = 2	-.03 (.41); n = 114
APOE rs7412	SDI	TT; CT;CC	0.04	0.65 (.19); n = 2	.19 (.48); n = 28	.01 (.40); n = 160
CHRNA4 rs6090387	SDI	CC;CG;GG	0.045	0.01 (.40); n = 112	.04 (.45); n = 69	.23 (.31); n = 9
SNAP25 rs6077690	SDI	AA;AT;TT	0.01	0.06 (.43); n = 62	.03 (.41); n = 104	.00 (.40); n = 24
CHRNA4 rs6090387	SDV	CC;CG;GG	0.03	-0.24 (.41); n = 112	-.17 (.44); n = 69	-.03 (.36); n = 9
HTR4 rs1862345	SDV	AA;AT;TT*	0.02	-0.18 (.44); n = 88	-.24 (.39); n = 84	-.13 (.43); n = 18
SLC6A3 rs6350	SDV	TT; CT;CC	0.03	0.27 (NA); n = 1	-.18 (.34); n = 23	-.21 (.43); n = 166
SNAP25 rs6077690	SDV	AA;AT;TT	0.02	-0.20 (.36); n = 62	-.22 (.47); n = 104	-.11 (.33); n = 24

NB = Neutral Bright; SBI = Single Bright Invalid; SDI = Single Dim Invalid; SDV = Single Dim Valid

Analyses were conducted with the log transformed RT using covariates; however unadjusted, raw RTs are shown for interpretability.

Positive slopes indicate increasing RT across approx. 24 trials of a given type (only correct trials were used).

* = unknown risk allele

The interaction between genotype and trial number (i.e., slope) was significantly associated with markers on nine genes. As can be seen in Table 2, two SNPs on *BDNF*, one on *DRD4*, two on *SLC5A7*, and two on *SLC6A3* were significantly associated with slopes for the Neutral Bright condition. One SNP each on *DRD4*, *IGF2*, *SLC5A7*, and *SLC6A3* was associated with slopes for the No Cue condition. One SNP on *APOE* and two SNPs on *SLC6A3* were significantly associated with slopes for the Single Bright Invalid condition. One SNP each on *APOE*, *CHRNA4*, and *SNAP25* was associated with the slopes for the Single Dim Invalid condition. Finally, one SNP each on *CHRNA4*, *HTR4*, *SLC6A3*, and *SNAP25* was associated with the Single Dim Valid condition. We will describe the direction of these significant findings in more detail by the neurotransmitter with which each gene is associated.

#### Acetylcholine

There were two associations with markers on *APOE*, both involving the rs7412 SNP. Rs7412 genotype groups differed significantly for slopes on the Single Bright Invalid condition, *F* (2, 3348) = 3.58, *p* = .03. Mean slope RT for those with zero risk alleles was 0.62 msec (*SD* = 0.02 msec), although there were only two individuals in this genotype group. Nevertheless, slope for those with one risk allele is in a different direction (0.02 msec; *SD* = 0.45 msec) from those with two risk alleles (-0.03 msec; *SD* = 0.42 msec). Only the two risk allele group is improving over the course of the task (over 180 total trials since trial number is used in analyses).

The *APOE* rs7412 genotype groups also differed significantly for slopes corresponding to the Single Dim Invalid condition, *F* (2, 3587) = 3.28, *p* = .04. Slope RT for those with zero risk alleles was 0.65 msec (*SD* = 0.19 msec). Slope RT for those with one risk allele was 0.19 msec (*SD* = 0.48 msec), and was 0.01 msec (*SD* = 0.40 msec) for those with two risk alleles. No individuals were improving over the course of the task for this condition, but the two-risk allele group showed the smallest increase in RT.

The *CHRNA4* gene likewise had a single marker (rs6090387) which was associated with two outcomes. For Single Dim Invalid (*F* [2, 3587] = 3.09, *p* = .045), those with no risk allele had a slope of 0.01 msec (*SD* = 0.40 msec), while those with two risk alleles had a slope of 0.23 msec (*SD* = 0.31 msec) and heterozygotes had slopes between these two extremes (*M* = 0.04 msec; *SD* = 0.45 msec). Likewise, Single Dim Valid (*F* [2, 3534] = 3.68, *p* = .03) had a significant linear trend in the same direction. Those with no risk alleles had a slope of -0.24 msec (*SD* = 0.41 msec), those with two risk alleles had slopes of -0.03 msec (*SD* = 0.36 msec), and heterozygotes had slopes between these two extremes, *M* = -0.17 msec; *SD* = 0.44 msec. This is a linear trend showing worse performance the more copies of the risk allele.

The *SLC5A7* gene had two SNPs significant for differential association with slopes by genotype. Rs3806536 was associated with Neutral Bright, *F* (2, 3577) = 3.50, *p* = .03. The risk allele for this SNP is unknown for attentional deficits. Slope RT for those with the AA genotype was -0.04 msec (*SD* = 0.32 msec). Slope for those with the AG genotype was -0.14 msec (*SD* = 0.41 msec) and -0.16 msec (*SD* = 0.33 msec) for those with the GG genotype. In other words, those with the AA genotype showed the least improvement over the course of the task. Rs4676169 was associated with the No Cue condition, *F* (2, 3778) = 4.69, *p* = .01 and with the Neutral Bright condition, *F* (2, 3577) = 3.70, *p* = .03. For the No Cue condition, those with the AA genotype has slopes of 0.04 msec (*SD* = 0.42 msec). Those with the AG genotype had slopes of -0.15 msec (*SD* = 0.47 msec) and those with the GG genotype had slopes of .03 msec (*SD* = .70 msec). For the Neutral Bright condition those with the AA genotype had slopes of 0.02 msec (*SD* = 0.40 msec). Those with the AG genotype had slopes of -0.15 msec (*SD* = 0.37 msec) and those with the GG genotype had slopes of -0.10 msec (*SD* = 0.33 msec). In both cases the heterozygotes improved in RT across the task. This pattern of results is termed heterozygous advantage.

The *SNAP25* gene is associated with all three neurotransmitters discussed in this paper. It had one SNP significant for differential association with two outcomes. Rs6077690 was significantly associated with slopes for Single Dim Invalid (*F* [2, 3587] = 4.39, *p* = .01) and Single Dim Valid, *F* (2, 3534) = 3.73, *p* = .02. For the association with Single Dim Invalid, the slope for those with the zero risk alleles the slope was 0.06 msec (*SD* = 0.43 msec). For those with one risk allele the slope was 0.03 msec (*SD* = 0.41 msec), and for those with two risk alleles the slopes was 0.00 msec (*SD* = 0.40 msec). For Single Dim Invalid, those with two risk alleles are not slowing over the course of the task while the other two genotype groups are. This is an overall linear trend. For Single Dim Valid, the linear trend is reversed so that those with two risk alleles are making the least improvement over the task. Slope for the zero risk allele group is -0.20 msec (*SD* = 0.36 msec). For the one risk allele group the slope is -0.22 msec (SD = 0.47 msec) and for the two risk allele group the slope is -0.11 msec (*SD* = 0.33 msec). This pattern of results is consistent with the risk allele as established in the literature.

#### Dopamine

The *DRD4* rs1800955 is significantly associated with slopes for the No Cue condition (*F* [2, 3778] = 3.43, *p* = .03) and for the Neutral Bright condition, *F* (2, 3577) = 3.27, *p* = .04. For the No Cue condition, slope RT for those with zero risk alleles was -0.01 msec (*SD* = 0.38 msec). Slope RT for those with one risk allele was -0.17 msec (*SD* = 0.45 msec), and was 0.08 msec (*SD* = 0.80 msec) for those with two risk alleles. Those with two risk alleles get slower over the course of the task while heterozygotes made the most improvement. While this is consistent with the risk allele as identified in the literature, the pattern of results also shows heterozygous advantage. For Neutral Bright, those with zero risk alleles had slopes of -0.11 msec (*SD* = 0.32 msec); those with one risk allele had slopes of -0.15 msec (*SD* = 0.38 msec); and those with two risk alleles had slopes of -0.03 msec (*SD* = 0.41 msec). This also shows a pattern consistent with heterozygous advantage.

The *IGF2* gene had one SNP significant for differential association with slopes by genotype. Rs734351 was associated with No Cue, *F* (2, 3778) = 3.39, *p* = .03. The risk allele for this SNP has not been established. Slope RT for those with the CC genotype was -0.05 msec (*SD* = 0.85 msec). Slope for heterozygotes was -0.01 msec (*SD* = 0.39 msec), and -0.14 (*SD* = 0.52 msec) for those with the TT genotype. This indicates that those with the TT genotype are getting the fastest over the course of the task.

*SLC6A*3 had six significant associations on four outcome measures. The *SLC6A3* VNTR was significantly associated with slopes for the No Cue condition, *F* (2, 3778) = 3.00, *p* = .049. Those with no risk alleles (i.e., the 5R/5R genotype) had slopes of -0.24 msec (*SD* = 0.60 msec). Heterozygotes had slopes of -0.04 msec (*SD* = 0.67 msec) and those with two risk alleles had slopes of -0.06 msec (*SD* = 0.45 msec). That is, those with two risk alleles are not getting faster over the course of the task for this condition as much as those with no risk alleles.

For rs2042449 there were also significant differences by genotype on slopes for the Single Bright Invalid condition, *F* (2, 3348) = 4.44, *p* = .01. The risk allele for this SNP has not been established; however, those with the CC genotype did not improve on RTs over the course of the task (their slope was 0.00 msec; *SD* = 0.46 msec) and those with the TT genotype slowed down over the course of the task (their slope was 0.08 msec; *SD* = 0.43 msec). Heterozygotes, however, did improve (their slope was -0.07 msec; *SD* = 0.36 msec). This pattern is heterozygous advantage.

Rs2617605 on *SLC6A3* showed significant differences by genotype for Single Bright Invalid (*F* [2, 3348] = 4.53, *p* = .01) and Neutral Bright, *F* (2, 3577) = 4.50, *p* = .01. Slopes for Single Bright Invalid were -0.01 msec (*SD* = 0.45 msec) for the zero risk allele group. Slopes for the two risk allele group were -0.03 msec (*SD* = 0.41 msec). Heterozygotes had slopes of 0.30 msec (*SD* = 0.52 msec), but there were only two participants in this group. This pattern is suggestive of a heterozygote disadvantage, but the heterozygote group is so small that it is difficult to say for sure. For Neutral Bright, those in the zero risk group had slopes of -0.07 msec (*SD* = 0.41 msec); heterozygotes had slopes of -0.03 msec (*SD* = 0.21 msec); and those in the two risk group had slopes of -0.13 msec (*SD* = 0.34 msec). For this condition results are opposite those expected for sustained attention.

Rs2937639 on *SLC6A3* was significant for slopes on Neutral Bright (*F* [2, 3577] = 3.82, *p* = .02). Slopes for those with zero risk alleles were 0.02 msec (*SD* = 0.37 msec) while those with one risk allele had slopes of -0.13 msec (*SD* = 0.38 msec) and those with two risk alleles had slopes of -0.14 msec (*SD* = 0.35 msec). This is the opposite direction of effect from established risk.

Rs6350 on *SLC6A3* was significant for slopes on Single Dim Valid, *F* (2, 3534) = 3.69, *p* = .03. Slopes for those with zero risk alleles were 0.27 msec (but there was only one participant in this genotype group). Nevertheless, those with one risk allele had slopes of -0.18 msec (*SD* = 0.34 msec) and those with two risk alleles had slopes of -0.21 msec (*SD* = 0.43 msec). Both genotype groups are getting faster, but the two risk allele group improved more.

#### Serotonin

The *BDNF* gene had two SNPs significant for differential association with slopes by genotype. Rs2203877 was associated with slopes for the Neutral Bright condition (*F* (2, 3577) = 3.13, *p* = .04) and rs6265 was also associated with slopes for the Neutral Bright condition, *F* (2, 3577) = 3.61, *p* = .03. For rs2203877, the risk allele is unknown. Slope RT for those with the CC genotype was -0.11 msec (*SD* = 0.37 msec). Slope for heterozygotes was -0.10 msec (*SD* = 0.41 msec), and slope for those with the TT genotype was -0.13 msec (*SD* = 0.30 msec). In this pattern, C appears to be the risk allele for this condition with a reflexive attention task. For rs6265 the zero risk allele group showed no improvement in RT over the course of the task (the slope was 0,00 msec (*SD* = 0.26 msec). For the one risk allele group slope was -0.12 msec (*SD* = 0.34 msec). For the two risk allele group, slope was -0.11 msec (SD = .39 msec). This pattern is opposite the expected based on the risk allele.

*HTR4* rs1862345 is significantly associated with slopes for the Single Dim Valid condition, *F* (2, 3534) = 4.17, *p* = .02. The risk allele is unknown. Those with the AA genotype had a slope of -0.18 msec (*SD* = 0.44). Those with the AT genotype had a slope of -0.24 msec (*SD* = 0.39 msec). Those with the TT genotype had a slope of -0.13 msec (*SD* = 0.43 msec). This pattern of results suggests heterozygous advantage.

## Discussion

Here we have shown that genetic markers influence slope variability on a reflexive attention task. Our findings are unlikely to be due solely to chance because (1) the models reported survived correction for multiple comparisons, (2) entering all genetic markers in a single model negates the need for correction for multiple comparisons within that model, and (3) they are biologically plausible. Regarding this third point, we tested genes involved in dopamine pathways (i.e., *COMT*, *DRD4*, *IGF2*, *MAOA*, *SLC6A3*, and *SNAP25*), which have been established as associated with some aspects of attention [33, 34]. Similarly, *BDNF*, *HTR4*, *MAOA* and *SNAP25* have been implicated in serotonin pathways, which have also been associated with attention [21], and *APOE*, *SLC5A7*, *CHRNA4*, and *SNAP25* have been connected to acetylcholine pathways, likewise associated with attention [35].

There were five of seven task outcomes that had three or more significantly associated genetic markers: Neutral Bright, No Cue, Single Bright Invalid, Single Dim Invalid, and Single Dim Valid. In the following paragraphs, we explore these findings including our observation that some of the significant findings can be explained by optimal dopamine levels (for findings showing heterozygous advantage on *DRD4* and *SLC6A3*) or for similar effects on the *SLC5A7* associated with acetylcholine and the *HTR4* gene associated with serotonin. This idea is explained later in more detail but essentially involves the idea that either too little or too much dopamine is problematic. Other findings might be explained by differences between this task and the task with which the gene (*APOE*, *BDNF*, *SLC6A3*, or *SNAP25*) was previously associated in the literature (because 7 of 14 associations with known risk allele in this report show an opposite direction of effect than that previously identified). Risk was established with attentional components in disorders such as Alzheimer’s disease [36], ADHD [37–39], depression [40, 41], memory impairment [42, 43], sustained attention deficits [21, 44–48].

In all, there were 21 association between 15 genetic markers (on nine genes) and slope on five outcome measures: Neutral Bright (7 associations), No Cue (4 associations), Single Bright Invalid (3 associations), Single Dim Invalid (3 associations), and Single Dim Valid (4 associations).

The Neutral Bright condition involves two equiluminant cues, one on each side of the display. Which side the target appears on is at chance. Participants might get slower over the course of the task for this condition more than for neutral dim if the bright cues become harder and harder to ignore over the course of the task. This might occur, for example, if the bright cues induce fatigue. Related ideas have been explored in studies dealing with visual fatigue associated with computer use [49–52], but it is not clear how these ideas relate to our task given that the tasks used in these studies are very different from ours.

The No Cue condition entails the target appearing after a variable delay from the previous trial. This condition might be especially difficult for participants who rely on a temporal warning that the target is about to appear, although (like the other conditions where validity is at chance) it also does not provide reliable location information.

The Single Bright Invalid condition presents a cue contralateral to where the target subsequently appears. It provides a temporal cue but the spatial information is misleading. Some participants may get worse at this condition over the course of the task if fatigue makes it harder and harder to ignore invalid cues. The Single Dim Invalid condition also involves a cue appearing contralateral to where the target will subsequently appear but uses a dim cue. We anticipated that dim cues (which are still clearly visible) might be more sensitive at distinguishing between genotypes.

The Single Dim Valid condition involves an ipsilateral cue preceding a target. Typically in this situation, participants are faster to respond than in a Single Dim Invalid or No Cue condition because attention is already in the correct location for responding to the target—there is no need to ignore the cue and attend only to the target. However, fatigue could make a participant respond more slowly over the course of the task. Once again, we will discuss the results by neurotransmitter.

### Acetylcholine

Of the three genes associated with acetylcholine, (*APOE*, *CHRNA4*, *SLC5A7*, and *SNAP25*), two were significantly associated with Single Dim Invalid, and one each with No Cue, Neutral Bright, Single Bright Invalid, and Single Dim Valid. In the case of *SLC5A7*, two different gens on the same gene were associated with the same outcome measure (Neutral Bright).

There were two associations between the *APOE* gene and slope outcomes. This gene is associated with the neurotransmitter acetylcholine and has been associated both with Alzheimer’s disease (which has attention-related symptoms) and with attention in healthy adults prior to any signs of Alzheimer’s disease [47]. *APOE* rs7412 was associated in our study with the Single Bright Invalid and Single Dim Invalid conditions, but in both cases it was associated in the opposite direction expected based on the literature regarding physiological impact of stress [53]. Since both luminances were affected, this implies that it is the validity (i.e., contralateral cues) that is giving some participants more difficulty as the task continues. *APOE*’s association with RT slope on the Single Bright Invalid condition is a replication of our finding in another sample using adult participants [30].

*CHRNA4* had a single SNP (rs6090387) that was associated with two dim outcomes, Single Dim Invalid and Single Dim Valid. In contrast with *APOE* (where both luminances were affected), here both validities are affected. This implies that it is the luminance (i.e., dim versus bright cues) that is giving some participants more difficulty as the task continues. We had anticipated that dim cues might give some participants more trouble because, while they are still clearly visible, they are not as salient and the bright cues [54]. The linear trend implies a gene-dose response very similar to a drug-dose response: the more copies of the risk allele, the stronger the effect.

*SLC5A7* had two SNPs (rs3806536 and rs4676169) that were associated with the Neutral Bright outcome. Rs4676169 was also associated with the No Cue condition. *SLC5A7* is a choline transporter involved in acetylcholine synthesis for cholinergic neurons [55]. As mentioned previously, this gene has been associated with attentional disorders [56–58], but the risk allele for neither SNP has been established. In our sample, heterozygotes tend to get faster over the course of the task for both SNPs and those with the AA genotype tend to either get slower (rs4676169) or show the smallest RT improvement (rs3806536). This suggests that those with the AA genotype on both SNPs are having trouble ignoring the bright cues for the Neutral Bright condition. There is no evidence that the SNPs are in linkage and so each result replicates the other result to indicate the importance of this gene to reflexive attention tasks. This contributes information for other researchers that the A allele on each SNP is the risk for the particular aspect of our reflexive attention task. Note that rs4676169 was also significantly associated with the No Cue condition in which participants must respond to a target with less warning because there are no cues that precede the target. While the risk allele has not been established for rs4676169, it is interesting to note that for both the No Cue and Neutral Bright conditions heterozygotes are performing faster as the task continues. Neither condition biases attention to one side or the other. That there is heterozygous advantage may indicate that there is an optimal level of acetylcholine for these conditions and that either too much or too little can have deleterious effects. This is a well-known pattern for dopamine and may apply to other neurotransmitters as well [59–62].

*SNAP25* also had a SNP (rs6077690) that was significantly associated with two outcome measures, Single Dim Invalid and Single Dim Valid. This gene acts in the presynaptic plasma membrane during neural transmission and is involved in the regulation of neurotransmitter release (including all three neurotransmitters we discuss: acetylcholine, dopamine, and serotonin). *SNAP25* has been associated with ADHD and symptoms of ADHD, such as impulsivity [63–66]. The association of this SNP with the two dim conditions is similar to that of *CHRNA4* and suggests that the relatively lower saliency might make this task progressively more difficult for some participants who are sensitive to luminance saliency in external cues. This seems to be the overall pattern in the cholinergic genes, that they are more sensitive to cue luminance such that dim cues (such as in the Single Dim Invalid) distinguish more between cholinergic genotypes.

### Dopamine

Of the three significant genes associated with dopamine (*DRD4*, *IGF2*, and *SLC6A3*), all three were associated with the No Cue condition, two were associated with the Neutral Bright condition, and one each with the Single Bright Invalid and Single Dim Invalid conditions.

*DRD4* is a type-four dopamine receptor and has been associated with ADHD and novelty seeking [67–73]. Our findings are consistent with those reported for rs1800955; participants with two copies of the risk allele (i.e., the CC group) either get slower over the course of the task (for the No Cue condition) or are not improving as much as the other genotype groups (for the Neutral Bright conditions). This might indicate decreased ability to ignore cues and attend only to targets. However, our findings are also consistent with heterozygous advantage which is a known characteristic of dopaminergic systems [59–62]. This suggests that too much or too little availability of dopamine can impair task performance.

*IGF2* had one SNP (rs734351) associated with an outcome. Like *DRD4*, *IGF2* is associated with the No Cue condition. The *IGF2* gene is an imprinted gene (i.e., expressed only from the allele inherited from the father). It is involved in growth and development generally, including cell differentiation of dopamine neurons during brain development [74]. Even though a risk allele for this SNP has not been established in the literature, those with the T allele get faster over the course of the task. Getting faster generally indicates learning. On the No Cue condition getting faster might indicate increased learning as well as the ability to attend to targets when there is no temporal warning that a target is about to appear.

*SLC6A3* is also known as the dopamine transporter, *DAT1*. The *SLC6A3* gene had six associations on the reflexive attention task. One of the findings (SNP rs2042449 with the No Cue condition) has an unknown risk allele. Our results are consistent with T as the risk allele (because those with the TT genotype get slower over the course of the task for this condition) or as consistent with heterozygous advantage (because heterozygotes get the fastest over the course of the task). The VNTR results are consistent with the direction of effects for sustained attention. However, the direction of effect for another SNP (rs2617605) appears to show opposite effects for our sustained attention task on the Neutral Bright and Single Bright Invalid conditions than those established for sustained attention. The two remaining associations (No Cue with rs2937639 and rs6350) are inconsistent with that established for sustained attention tasks. This is an important and useful finding because it indicates that reflexive attention might have fundamentally different associations than sustained attention.

As was mentioned above, *SNAP25* is associated with all three neurotransmitters we discuss. *SNAP25* was associated with Single Dim Invalid and Single Dim Valid conditions. As such, it shares association with one other dopaminergic gene. *SLC6A3* has one SNP (rs6350) associated with Single Dim Valid.

Overall the pattern for dopamine is similar to that for acetylcholine, showing sensitivity to luminance. However, for dopamine it is bright cues that differentiate more between genotypes. It is notable that there were five significant associations with bright cues and only one significant association with dim cues for these genetic markers.

### Serotonin

Of the two significant genes associated with serotonin (*BDNF* and *HTR4*), one was associated with Neutral Bright and one with Single Dim Valid. The *BDNF* gene had two associations with RT slope outcomes for the Neutral Bright condition. The *BDNF* gene promotes differentiation, axonal growth, pathfinding, and dendritic growth during development. It is associated with the neurotransmitter serotonin, which is active during development [17]. The risk allele for rs2203877 is unknown, but we would identify the risk allele as C. Another SNP (rs6265) on the BDNF gene has a known risk allele for sustained attention, and yet our results show an opposite pattern than that established in the literature. There is no evidence for linkage between these two SNPs and so, as with *SLC5A7*, each replicates the other result to confirm the importance of *BDNF* in reflexive attention. That a gene involved in brain development is associated a reflexive attention task in childhood suggests that there may be a somewhat inadequate serotonergic system in some children with attentional deficits.

*HTR4* had one SNP (rs1862345) that was significantly associated with slope for the Single Bright Invalid condition. *HTR4* codes for a serotonin receptor. Reduced receptor number might decrease neural transmission even if there is otherwise enough serotonin available. However, somehow too many receptors is also nonideal. Perhaps too many receptors interfere with other actions necessary for neural transmission. Heterozygous disadvantage can happen when there is an optimal amount of a gene product (receptors) for performance and too much or too little can interfere with a particular outcome. One neurotransmitter with optimal level effects is dopamine [59–62], but the effect may occur for other gene products as well.

As was mentioned above, *SNAP25* is associated with all three neurotransmitters we discuss. *SNAP25* was associated with Single Dim Valid conditions as were *CHRNA4* in the acetylcholine system, *SLC6A3* in the dopamine system, and *HTR4* in the serotonin system. SNAP25 was also associated with Single Dim Invalid. This seems to indicate that dim cue conditions are especially sensitive at detecting issues with neurotransmitter release.

## Conclusion

Overall our results show that Neutral Bright cues and No Cue conditions (conditions which do not bias attention to one side or the other) distinguish well by genotype for genes associated with all three neurotransmitters. Conditions with dim cues also seem sensitive to genetic differences (also for all three neurotransmitters). Other patterns in the data suggest that there are different risk alleles for reflexive attention tasks than for sustained attention tasks and that there are optimal levels of availability of neurotransmitters (evidenced by heterozygous advantage). The genes that had significant markers on them were *APOE* (1 marker), *BDNF* (2 markers), *CHRNA4* (1 marker), *DRD4* (1 marker), *IGF2* (1 marker), *SLC5A7* (2 markers), SLC6A3 (5 markers), and *SNAP25* (1 marker). The genes are associated with all three neurotransmitters we tested (acetylcholine, dopamine, and serotonin). All these neurotransmitters have been established to have association with sustained attention.

As with all genetic association studies, our findings will need replication. This is especially true because genetic association studies with reflexive attention tasks are rare. However, we have shown partial replication and extension of previous results using sustained attention to reflexive attention tasks. In some cased the direction of effects were the same as for disorders commonly associated with sustained attention deficits, but more often the results were in the opposite direction. This indicates the additional usefulness of reflexive attention tasks in studying attentional deficits. Studies should not be limited to sustained attention tasks when measuring attention. We have also contributed by making associations with SNPs for which the direction of effect was not known even though they are on genes associated with attention. This information is useful and warrants replication attempts. Ultimately, the present results add further evidence that genetics do contribute to slope variability in reflexive attention tasks. Further research into the genetics of attention has the potential to improve intervention for those with attentional deficits by targeting for intervention the specific types of tasks that give them trouble.
